# Metabolic Reprogramming towards Aerobic Glycolysis Correlates with Greater Proliferative Ability and Resistance to Metabolic Inhibition in CD8 versus CD4 T Cells

**DOI:** 10.1371/journal.pone.0104104

**Published:** 2014-08-04

**Authors:** Yilin Cao, Jeffrey C. Rathmell, Andrew N. Macintyre

**Affiliations:** Department of Pharmacology and Cancer Biology, Department of Immunology, Sarah W. Stedman Center for Nutrition and Metabolism, Duke University, Durham, NC, United States of America; University Hospital of Heidelberg, Germany

## Abstract

T lymphocytes (T cells) undergo metabolic reprogramming after activation to provide energy and biosynthetic materials for growth, proliferation and differentiation. Distinct T cell subsets, however, adopt metabolic programs specific to support their needs. As CD4 T cells coordinate adaptive immune responses while CD8 T cells become cytotoxic effectors, we compared activation-induced proliferation and metabolic reprogramming of these subsets. Resting CD4 and CD8 T cells were metabolically similar and used a predominantly oxidative metabolism. Following activation CD8 T cells proliferated more rapidly. Stimulation led both CD4 and CD8 T cells to sharply increase glucose metabolism and adopt aerobic glycolysis as a primary metabolic program. Activated CD4 T cells, however, remained more oxidative and had greater maximal respiratory capacity than activated CD8 T cells. CD4 T cells were also associated with greater levels of ROS and increased mitochondrial content, irrespective of the activation context. CD8 cells were better able, however, to oxidize glutamine as an alternative fuel source. The more glycolytic metabolism of activated CD8 T cells correlated with increased capacity for growth and proliferation, along with reduced sensitivity of cell growth to metabolic inhibition. These specific metabolic programs may promote greater growth and proliferation of CD8 T cells and enhance survival in diverse nutrient conditions.

## Introduction

Prior to activation, T lymphocytes (T cells) are quiescent and use only low rates of metabolism to fuel migration and homeostatic proliferation. Once activated by antigen presenting cells, CD4 and CD8 T cells proliferate rapidly and undergo environmentally directed differentiation into diverse effector cell populations. These effector cells optimize the immune response for specific pathogenic challenges. Activated CD4 T cells can differentiate into T helper (Th) subpopulations to combat bacterial or fungal infections, while activated CD8 T cells can differentiate into cytotoxic T cells to combat viral infections. Activation and the transition from naïve to effector lymphocyte greatly alters cellular metabolic demands, as cells require both ATP and biosynthetic components to fuel growth, cell division, migration, and subset differentiation [1]. Activation-induced metabolic reprogramming may be important to enable effector populations to fulfill their specific immunological roles, as different T cell populations have been reported to adopt distinct metabolic programs. *In vitro* generated Th CD4 T cells are highly glycolytic, performing high rates of glycolysis and minimal fatty acid oxidation. In contrast, inducible CD4 regulatory T cells exhibit low rates of glucose uptake, with high rates of fatty acid oxidation [2]–[4]. Similarly, CD8 cytotoxic T cells have been shown to adopt a highly glycolytic metabolism [5], [6], but transition to fatty acid oxidation as memory cells [7].

Activation-induced metabolic reprogramming events include elevated expression of metabolite transporters [8]–[12]; isozyme switching and elevated production of glycolytic enzymes [3], [13], [14]; increased glycolytic flux; and increased rates of oxidative phosphorylation [3], [9], [15]. The net result of early lymphocyte metabolic reprogramming is a switch towards a highly glycolytic metabolism, wherein cells undertake high rates of glycolysis but perform comparatively low rates of oxidative phosphorylation (OXPHOS), preferentially secreting glucose-liberated carbon as lactate. This metabolic strategy is reminiscent of the aerobic glycolysis phenotype frequently observed in cancer cells [16], and supports both biosynthesis and proliferation by maintaining ATP and NAD+ levels, restricting reactive oxygen species production, and increasing biosynthetic flexibility [17]. Recently, we examined mice that had a T cell specific deletion of the glucose transporter Glut1, the major activation-induced glucose transporter in both CD4 and CD8 T cells. Naïve CD4 and CD8 T cells in these mice occurred at expected ratios and numbers. Surprisingly, however, while CD4 Th cells were significantly affected by Glut1 deletion, CD8 cytotoxic T cells were not [12]. These data suggest that CD4 and CD8 cells adopt different metabolic programs following activation. Indeed, it is still unclear how activation-induced metabolic rewiring enables CD4 and CD8 T cells to perform different immunological functions or support their distinct biological characteristics.

Here, we compare the metabolic programs of CD4 and CD8 lymphocytes both *ex vivo* and following activation. We demonstrate that activated CD4 lymphocytes have greater mitochondrial mass and are consistently more oxidative, while activated CD8s preferentially adopt a more glycolytic metabolism. This difference is associated with the faster growth and proliferative rates of activated CD8 T cells, along with reduced sensitivity of cell growth to metabolic inhibition.

## Results

### Stimulated CD8 T cells grow and proliferate faster than CD4 T cells

**Figure 1:**
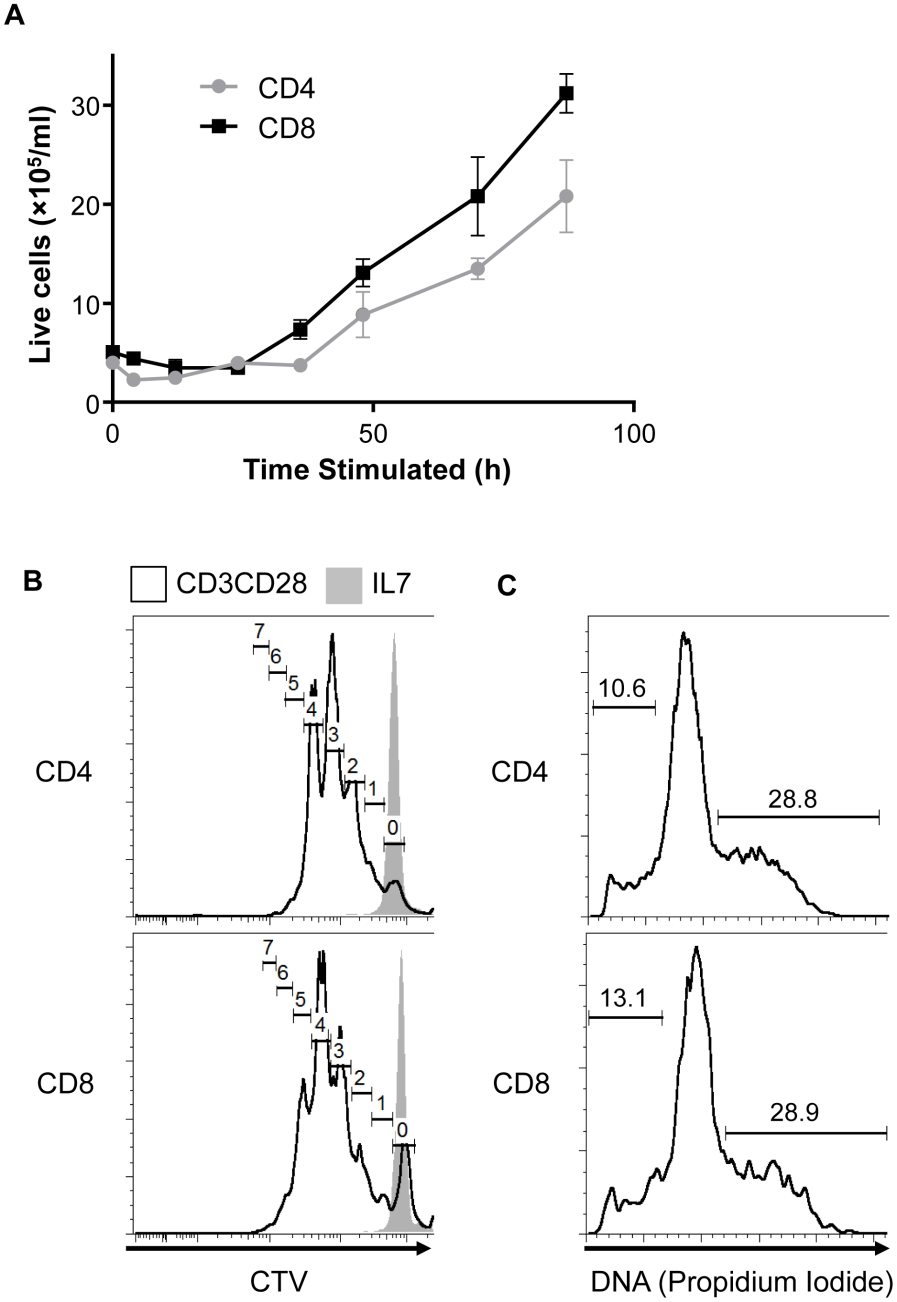
CD8 T cells proliferate faster than CD4 T cells. **(A)** CD4 and CD8 T cells were stimulated with anti-CD3 anti-CD28 and the number of viable cells was counted as shown. (**B**) CD4 and CD8 T cells were labeled with CellTrace Violet and then activated with anti-CD3 anti-CD28 for 3 days. CellTrace Violet dilution was measured by flow cytometry. Gates show generation numbers. (**C**) CD4 and CD8 T cells were activated for 3 days with anti-CD3 anti-CD28 and propidium iodide staining was then used to detect cellular DNA content by flow cytometry. Small gates show percentage of non-doublet cells with sub-diploid DNA content, large gates show percentage of non-doublet cells in G_2_ and S cycle phase. (**A–C**) All data are representative from a minimum of (**a**) two or (**b**, **c**) three independent experiments.

CD4 T cells are activated *in vivo* by stimulation of the TCR by MHC Class II presenting cognate antigen, while the TCR on CD8 T cells binds antigen presented on MHC Class I. These distinct ligands signal through the CD3 components of the TCR complex together with costimulatory molecules such as CD28 to trigger metabolic reprogramming, growth, proliferation, and differentiation [18]. To directly compare CD4 and CD8 activation with same ligand *in vitro,* CD4 and CD8 T cells were isolated from the spleen and lymph nodes of C57BL/6 mice and then stimulated with plate-bound antibodies against CD3 and CD28 in the presence of IL-2. CD4 cells were depleted of CD25+ natural regulatory T cells prior to stimulation. After a 24 h lag period, the viable cell number of CD8 T cells began to rapidly increase. CD4 T cells, however, accumulated more slowly (**Fig. 1A**). To test if cellular accumulation reflected differences in proliferation rate, purified CD4 and CD8 cells were labeled with the proliferation indicator dye CellTrace Violet (CTV) and then stimulated as before. After 72 h, CD3 and CD28 stimulation CD8 T cells showed a greater dilution of CellTrace Violet in comparison to CD4 cells, indicating increased proliferation in the CD8 cells (**Fig. 1B**). Propidium iodide assessment of cell cycle status indicated that similar percentage of activated CD4 and CD8 T cells were in the S and G_2_ phases of the cell cycle after 72 h activation (**Fig. 1C**). A lower frequency of CD4 T cells had undergone apoptosis, as indicated by sub-diploid DNA content (**Fig. 1C**). Collectively, these data show that CD8 T cells start proliferating earlier and faster than CD4 cells, but that after 72 h the proliferative differences between the cell types are minor.

**Figure 2:**
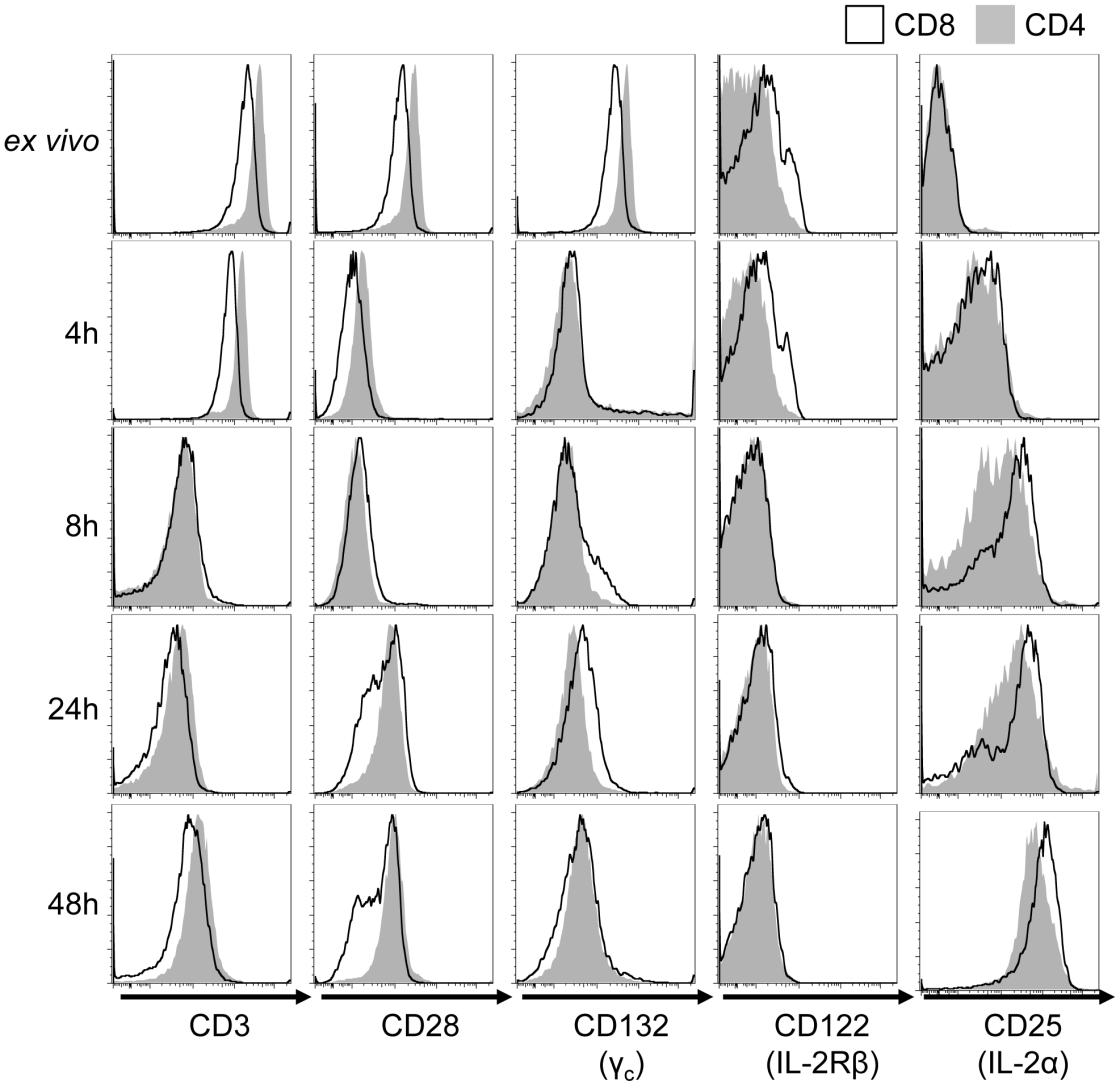
CD4 and CD8 T cells express similar levels of CD3 and IL-2 receptor. Isolated CD4 and CD8 T cells were stimulated on anti-CD3 anti-CD28 coated plates in the presence of IL-2 for the times shown. Surface expression of CD3, CD28, CD25, CD132 and CD122 were measured by flow cytometry. Data are representative from two independent experiments.

The difference in proliferative response between CD4 and CD8 cells could arise from reduced surface expression of CD3, CD28, or IL-2 receptor on naïve CD4 T cells. However, resting CD4 T cells were found to express slightly more CD3 and CD28 on their surface in comparison to CD8 T cells (**Fig. 2**). The expression of the intermediate-affinity IL-2 receptor heterodimer chains (γ_c_: CD132; IL-2Rβ: CD122) was similar between resting CD4 and CD8 cells; however, following stimulation CD8 T cells showed more rapid expression of CD25 (IL-2Rα: CD25), which pairs with γ_c_ and IL-2Rβ to form a high-affinity heterotrimeric IL-2 receptor [19]–[21] (**Fig. 2**).

### CD4 and CD8 T cells both undergo metabolic reprogramming, but CD4 T cells increase oxidative metabolism to a greater extent than CD8 T cells

**Figure 3:**
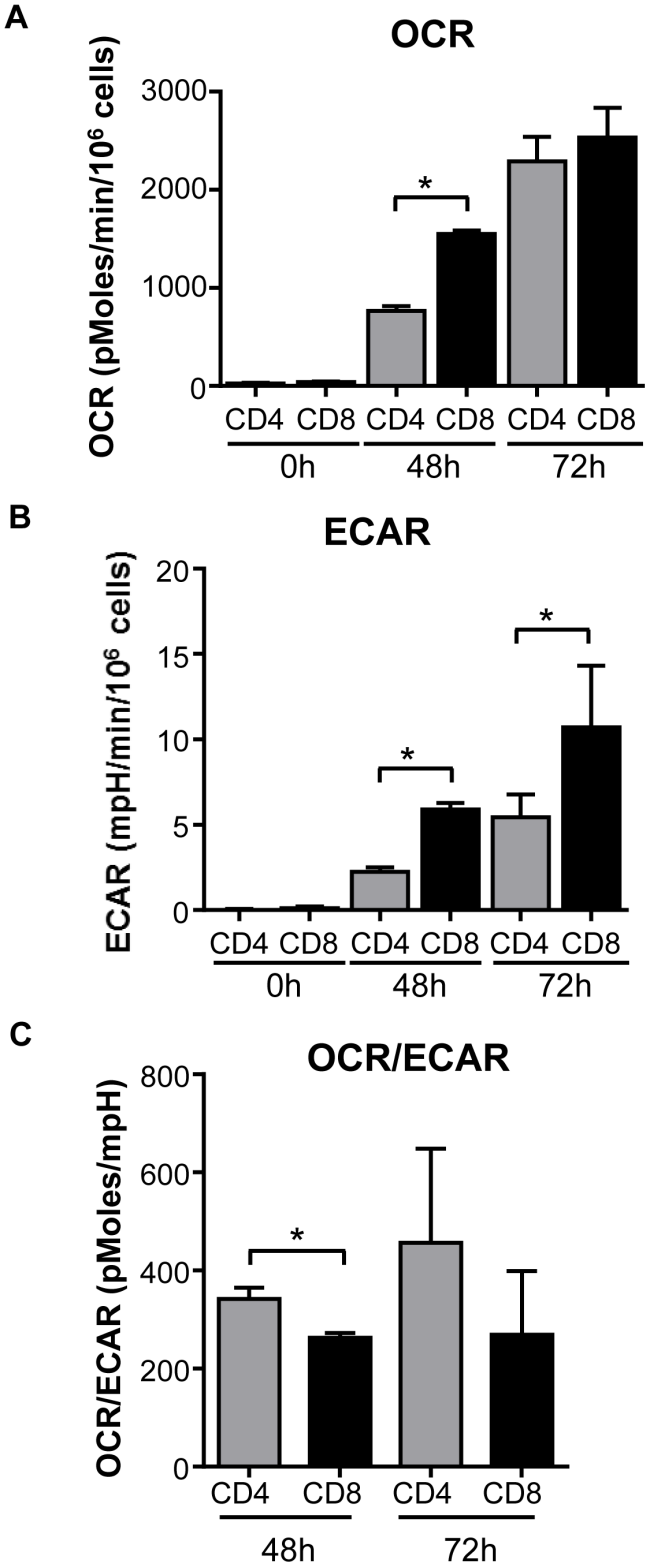
CD4 T cells are more oxidative than CD8 T cells. CD4 and CD8 T cells were activated for 48 or 72-CD3 anti-CD28 and then (**A**) oxygen consumption rate (OCR) and (**B**) extracellular acidification rate (ECAR) were measured in comparison to CD4 and CD8 T cells isolated directly *ex vivo.* Cells were activated in regular culture media, and flux analysis was performed in media containing 10 mM D-glucose, 10 mM L-glutamine, and 10 mM sodium pyruvate. (**C**) OCR/ECAR ratio was calculated for 48 and 72 h stimulated cells. Data show mean ± standard deviation from representative experiment. Data are representative from a minimum of two independent experiments.

Activated T cells increase metabolic pathways to support the energetic and biosynthetic demands of cell growth, proliferation, and gain of effector function [1]. CD4 and CD8 T cells activated through the TCR received the same initial signals yet proliferated at different rates. To determine if CD4 and CD8 T cells induced the same or distinct metabolic programs, extracellular flux analyses were performed to measure glycolysis and oxygen consumption. In the presence of abundant glucose, pyruvate and glutamine, resting CD4 and CD8 T cells *ex vivo* exhibit only a low oxygen consumption rate (OCR) and extra cellular acidification rate (ECAR) (**Fig. 3A, B**). Following activation, CD4 and CD8 cells dramatically increased both OCR and ECAR, although CD8 cells showed a stronger preference for aerobic glycolysis, as shown by the lower OCR/ECAR ratio (**Fig. 3C**). After 72 h of stimulation, both populations exhibited similar OCR, however, the ECAR for CD8 cells remained higher (**Fig. 3B**). As a result, CD8 T cells maintained a lower ratio of OCR to ECAR (OCR/ECAR) than CD4 cells. Hence, while both CD4 and CD8 T cells switch towards aerobic glycolysis when activated, CD4 T cells retain a proportionally more oxidative metabolism than CD8 T cells, which were relatively more glycolytic.

**Figure 4:**
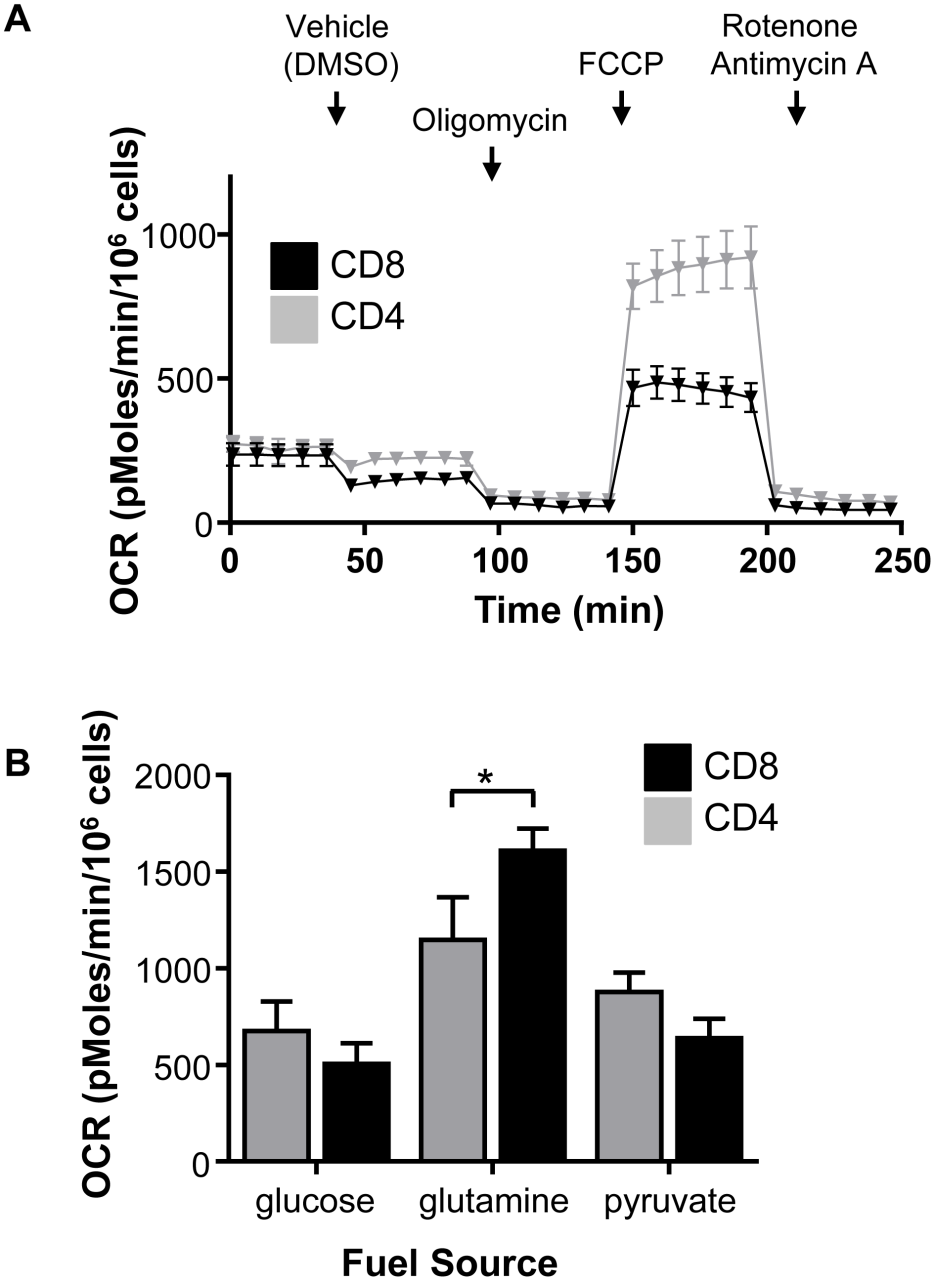
CD4 T cells have a higher respiratory capacity and are more oxidative than CD8 T cells. **(A, B)** CD4 and CD8 T cells were activated for 72-CD3 anti-CD28 and then compared to CD4 and CD8 T cells isolated directly *ex vivo*. (**A**) Real time changes in oxygen consumption rate (OCR) were measured in response to addition of the indicated compounds. OCR analysis was performed in media containing 10 mM D-glucose, 10 mM L-glutamine, and 10 mM sodium pyruvate. (**B**) CD4 and CD8 T cells were activated for 72 h with anti-CD3 anti-CD28 and then OCR was determined by extracellular flux analysis. Cells were activated in regular culture media, and flux analysis was performed in media supplemented with 10 mM D-glucose, 10 mM L-glutamine, or 10 mM sodium pyruvate as indicated. (**A–C**) Data shown are representative mean ± standard deviation from two independent experiments.

Oxidative metabolism and metabolic flexibility are constrained by mitochondrial oxidative capacity. The maximal respiratory capacity of stimulated CD4 and CD8 T cells were therefore compared. OCR was measured under basal conditions and following the addition of oligomycin (to reduce OCR to baseline by inhibiting ATP synthase), FCCP (to maximize OCR by uncoupling the electron transport chain (ETC) from ATP synthesis), and rotenone plus antimycin A (to block the ETC by inhibiting ETC complexes I and III). The maximal respiratory capacity after uncoupling by FCCP was significantly higher in activated CD4 T cells than in activated CD8 T cells (**Fig. 4A**). Activated CD4 T cells therefore have a substantially greater capacity for oxidative metabolism than similarly stimulated CD8 cells.

CD4 and CD8 T cells were next examined to determine the ability of cells to utilize distinct metabolic fuels that may reflect differential degrees of metabolic flexibility. Nutrients were provided individually to previously activated CD4 and CD8 T cells and OCR was measured. Strikingly, CD8 T cells responded to glutamine with a higher OCR than CD4 T cells (**Fig. 4B**). These data indicate that while CD4 T cells have a higher oxidative capacity when glucose is the primary fuel, CD8 T cells have greater capacity for glutamine oxidation. A trend towards higher OCR for CD4 T cells was observed in the presence of glucose and pyruvate, but these differences were not significant. Thus, while CD4 T cells appear to have selectively increased capacity for mitochondrial respiration of glucose, both CD4 and CD8 T cells can oxidize alternative fuels if required.

### Activation induces similar molecular changes to reprogram metabolism in CD4 and CD8 T cells

**Figure 5:**
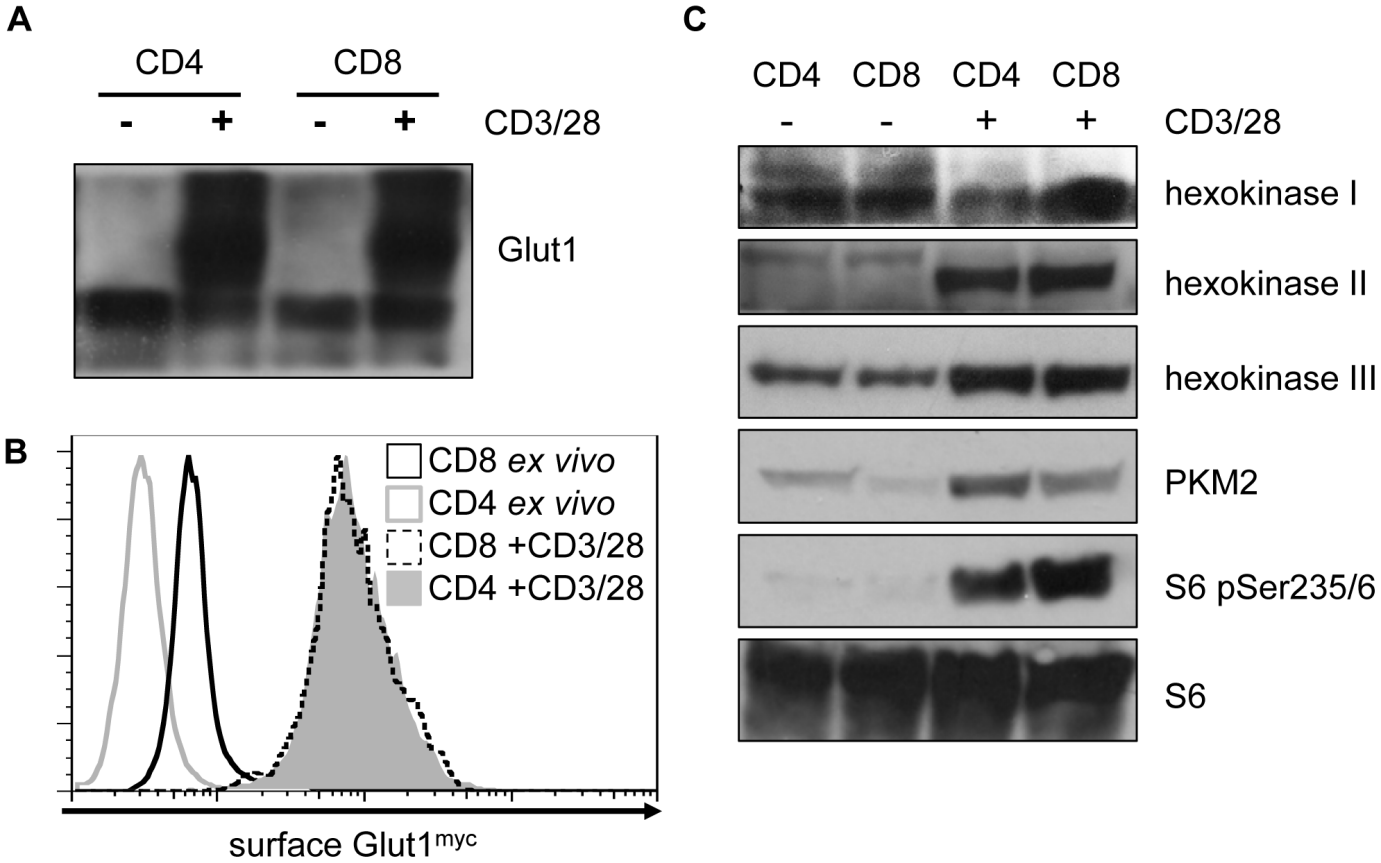
Following activation CD4 and CD8 T cells undergo similar glycolytic reprogramming events. CD4 and CD8 T cells were activated for 72-CD3 anti-CD28 in the presence of IL-2. CD4 and CD8 cells were isolated directly *ex vivo* for comparison and then all cells were lysed and analyzed by immunoblot for expression of (**A**) Glut1, or (**C**) hexokinase, PKM2, cytochrome C, Ser235/236 phosphorylated small ribosomal subunit S6, and total S6. (**B**) CD4 and CD8 Glut1^myc/myc^ T cells expressing myc tagged Glut1 at endogenous levels were isolated and activated for 48 h with anti-CD3 anti-CD28 in the absence of IL-2. Surface expression of Glut1^myc^ was then measured by flow cytometry. Glut1^myc/myc^ T cells analyzed directly *ex vivo* were used as a control. (**A–C**) CD4+CD25+ depletion was not performed prior to stimulation. All data are representative of a minimum of three independent experiments.

Lymphocyte activation triggers a dramatic increase in glucose uptake and glycolysis. This is facilitated by increased surface expression of glucose transporters, including Glut1 [12], [22], and also by increased hexokinase expression and activity [14], [23]. Following activation CD4 and CD8 T cells exhibited dramatic increases in the expression of total Glut1 protein (**Fig. 5A**). No significant differences were observed in the total levels of Glut1 protein between CD4 and CD8 T cells. Glut1 trafficking is highly regulated and cell surface Glut1 expression was next measured flow cytometrically using Myc-epitope tagged Glut1 knock-in T cells [2] (**Fig. 5B** **)**. Resting CD8 T cells had higher basal cell surface expression of Glut1. After 48 h activation, however, no differences in total or surface Glut1 levels could be detected between CD4 and CD8 T cells (**Fig. 5B**).

Following uptake, glucose is phosphorylated by hexokinase and can be metabolized through glycolysis. T cell activation resulted in a modest drop in hexokinase I (HKI) expression in CD4 T cells and an increase in HKI expression in CD8 T cells. In both cell types HKII and HKIII were strongly induced by CD3/CD28 activation, with both activated CD4 and activated CD8 T cells exhibiting similar total levels of HKII and HKIII (**Fig. 5C**). The M2 isoform of Pyruvate Kinase (PKM2) is a key glycolytic enzyme in cancer cells [24]. We examined naïve and activated lymphocytes for the expression of PKM2 (**Fig. 5C**). PKM2 expression was increased in both CD4 and CD8 T cells following activation. Of note, activated CD4 T cells had a greater expression of PKM2 than CD8 T cells activated under the same conditions. mTOR is a key regulator of T cell glycolysis [25] and this pathway appeared to be elevated in CD8 T cells relative to CD4 T cells based on increased levels of the downstream effector of mTOR signaling, phospho-S6 (**Fig. 5C**). Together, these data suggest that the metabolic reprogramming events that drive elevated glucose uptake and glycolysis in activated CD4 and CD8 T cells are generally similar, although not identical.

**Figure 6:**
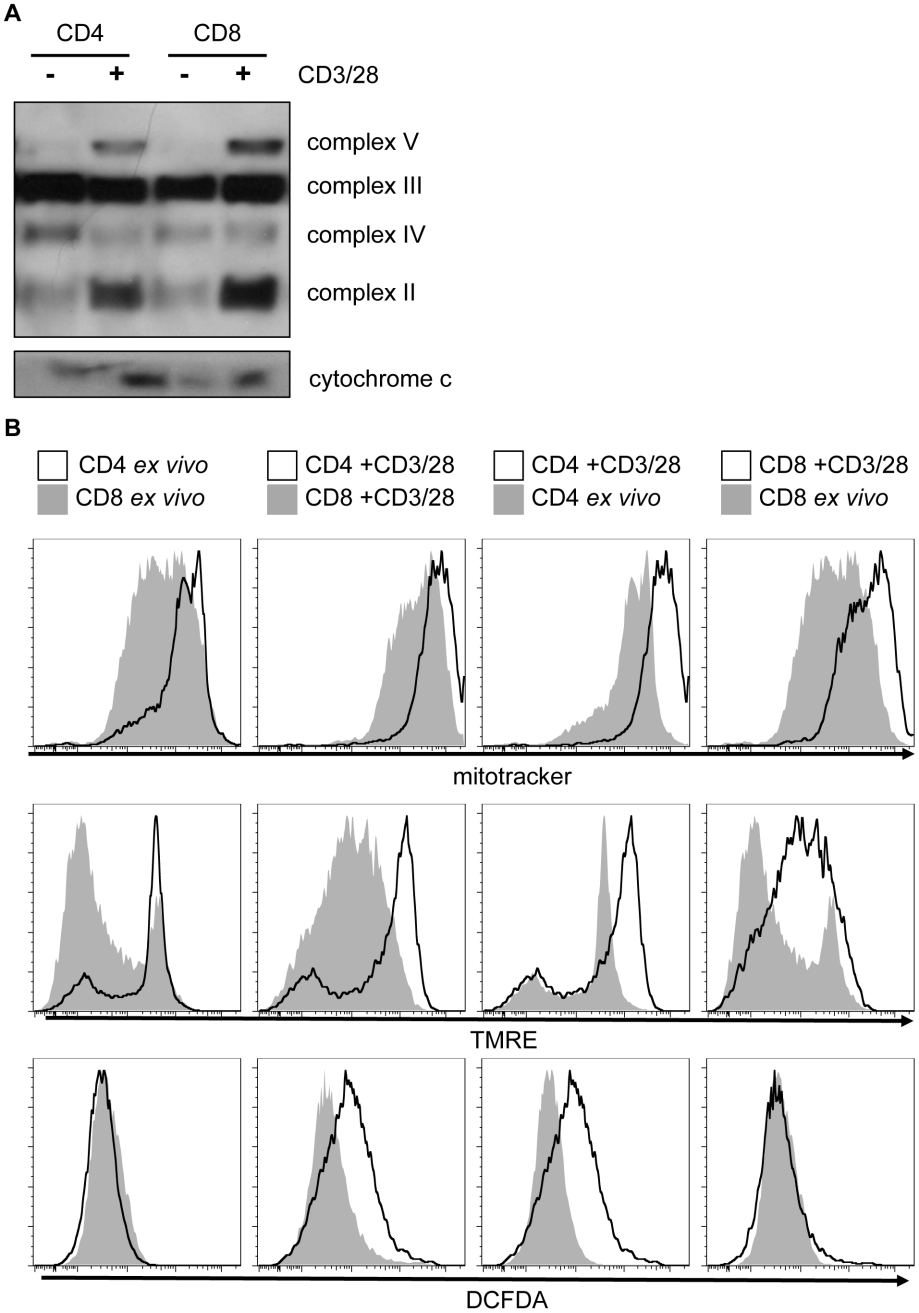
CD4 T cells have greater mitochondrial content and reactive oxygen species than CD8 T cells. CD4 and CD8 T cells were activated for 72-CD3 anti-CD28. CD4 and CD8 cells were isolated directly *ex vivo* for comparison and then cells were (**A**) lysed and analyzed by immunoblot mitochondrial OXPHOS complex subunits; or (**B**) stained with mitotracker to measure mitochondrial content, TMRE, to measure mitochondrial membrane potential, or DCF, to measure reactive oxygen species. Mitotracker and DCF staining were quantified by flow cytometry and histograms are overlaid as indicated. (**A**) CD4+CD25+ depletion was not performed prior to stimulation. (**A–B**) All data are representative of a minimum of three experiments.

To examine the mechanism of preferentially increased OCR and maximal respiratory capacity after CD4 T cell activation, mitochondrial mass and function were next assessed in each T cell lineage. The relative protein expression of cytochrome c and the OXPHOS mitochondrial complexes were examined in naïve and stimulated CD4 and CD8 lymphocytes (**Fig. 6A**). Electron transport complexes were detected using an antibody mix that detects key component proteins from each complex. Activation of either CD4 or CD8 T cells resulted in a dramatic increase in expression of cytochrome c, and complexes II and V (**Fig. 6A**). Complex III and IV levels stayed constant or decreased, while complex I was not measured. No consistent differences were observed between CD4 and CD8 T cells. In contrast, flow cytometric analysis of CD4 and CD8 T cells labeled with a mitochondrial membrane dye, Mitotracker, showed higher staining in CD4 T cells that increased further with stimulation. Mitotracker did not increase in activated CD8 T cells and instead appeared to lead to an increased population of cells with lower mitochondrial lipid content (**Fig. 6B** **top**). CD4 T cells also exhibited higher staining with the potentiometric dye TMRE, both *ex vivo* and after activation. This suggests that in addition to elevated mitochondrial mass, CD4 cells have a greater mitochondrial membrane potential [26] (**Fig. 6B** **middle**). Mitochondrial activity can lead to production of reactive oxygen species (ROS) and we also found that resting CD4 T cells had higher levels of staining with the ROS-sensitive dye, DCFDA, and that ROS further increased upon activation (**Fig. 6B** **bottom**). Together, our data show that while both CD4 and CD8 T cells reprogram their metabolism following activation, CD8 T cells preferentially adopt a highly glycolytic metabolism, while CD4 cells have greater degree of mitochondrial content and activity.

### Activation context fine-tunes metabolic reprogramming

**Figure 7:**
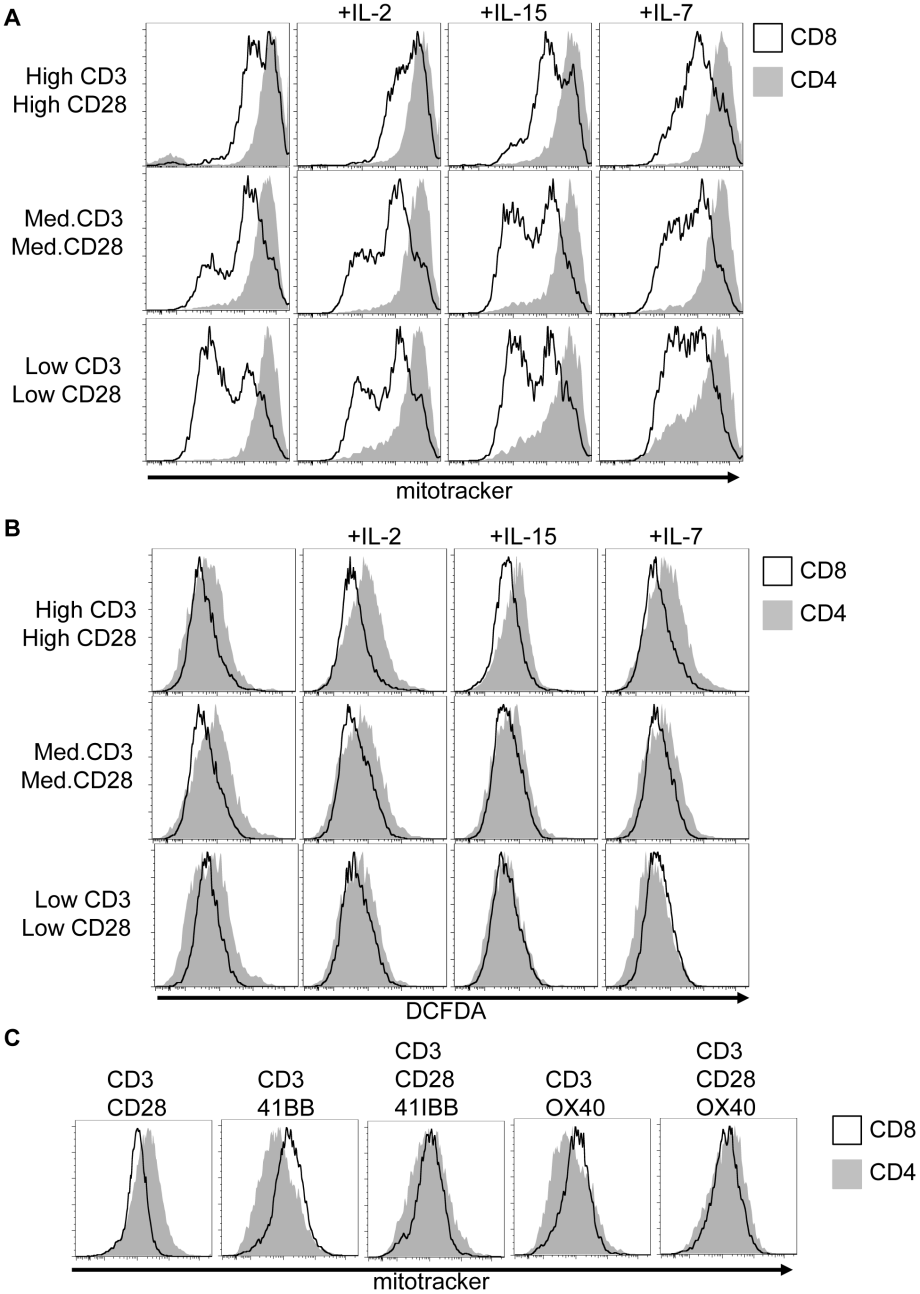
Activation context fine-tunes metabolic reprogramming. (A, B) Isolated CD4 and CD8 T cells were stimulated for 72 h on plates coated with high (10 µg/ml), medium (2.5 µg/ml) or low (0.5 µg/ml) doses of anti-CD3 and anti-CD28, plus 20 ng/ml IL-2, 10 ng/ml IL-15, or 10 ng/ml IL-7 as indicated. Shown are (**A**) mitotracker staining of mitochondrial mass, and (**B**) DCFDA measurement of reactive oxygen species. (**C**) Isolated CD4 and CD8 T cells were stimulated for 72 h on plates coated with 10 µg/ml anti-CD3, anti-CD28, anti-41BB, or anti OX40, as indicated. Mitotracker staining was quantified by flow cytometry. (**A–C**) All data are representative of a minimum of two experiments.

When stimulated with a high dose of anti-CD3, anti-CD28 in the presence of IL-2, CD4 T cells have greater mitochondrial mass and produce more ROS than similarly stimulated CD8 T cells (**Fig. 6B**). To determine the impact of signal strength and cytokine environment on these differences, CD4 and CD8 T cells were stimulated for 72 h with low (0.5 µg/ml), medium (2.5 µg/ml) or high (10 µg/ml) doses of anti-CD3 and anti-CD28 either alone or in the presence of IL-7, IL-15, or IL-2. Each of these cytokines provide stimulation via receptors comprised of γ_c_ plus unique accessory chains; however, they activate PI3K/Akt signaling to different extents, stimulate different JAK/STAT cascades and have different immunoregulatory functions [27]. Irrespective of the stimulation context, CD4 T cells exhibited higher mitochondrial mass than CD8 T cells (**Fig. 7A**), with CD8 T cell mitochondrial mass exhibiting a greater sensitivity to anti-CD3/CD28 concentration. ROS production in both CD4 and CD8 T cells was sensitive to CD3/CD28 stimulation strength, with DCFDA staining increasing as stimuli concentration was increased. At high and medium dose anti-CD3/CD28, CD4 T cells exhibited greater ROS staining in comparison to CD8 cells irrespective of cytokine; however, at low dose anti-CD3/CD28 there was little or no difference in ROS between populations (**Fig. 7B**).

T cell priming is refined by both co-stimulatory and co-inhibitory signals from co-receptors. To examine if the metabolic differences between CD4 and CD8 T cells were influenced by co-stimulatory receptors beyond CD28, T cells were activated in the presence of anti-CD3, anti-CD28, plus either anti-4-1BB (CD137) or anti-OX40 (CD134). 4-1BB and OX40 are activation-induced co-receptors that can enhance T cell cytokine release, proliferation, and survival. Activated CD4 and CD8 cells express both receptors; however, 4-1BB stimulation acts chiefly on CD8 cells [28], [29], while OX40 has primarily been described as regulating CD4 cells [30]. High-dose anti-CD3/CD28 plus either anti-4-1BB or anti-OX40 led to activated CD4 and CD8 T cells that had similar mitochondrial content. OX40 or 4-1BB co-stimulation in the absence of CD28 stimulation led to CD8 T cells having higher mitochondrial mass than similarly stimulated CD4 cells (**Fig. 7C**).

Collectively, these data demonstrate that while activated CD4 and CD8 T cells are metabolically different, activation-induced metabolic reprogramming in each population is fine tuned by both co-stimulatory and cytokine signals. Metabolic rewiring in both CD4 and CD8 T cells is, therefore, in part dictated by the activation environment but is also distinct due to cell-intrinsic differences between these cell populations.

### CD4 and CD8 T cells are reliant on glycolysis and oxidative metabolism for survival, growth and proliferation

**Figure 8:**
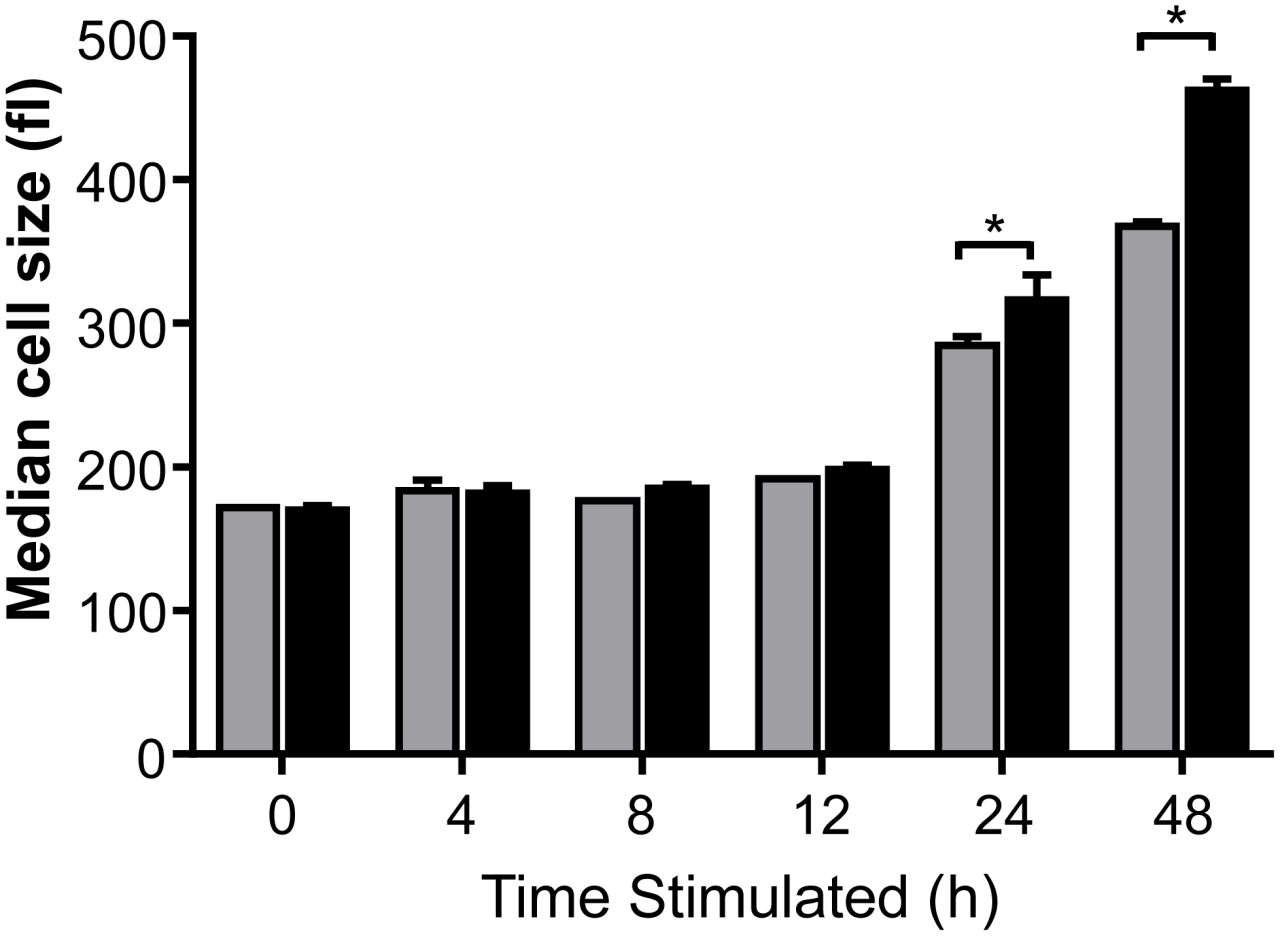
CD8 T cells grow more rapidly than CD4 T cells. Isolated CD4 and CD8 T cells were activated with anti-CD3 anti-CD28 and median cell volume in fl was measured by Coulter counter at the timepoints indicated. Data show median ± standard deviation of technical triplicates. Data shown are representative from three independent experiments.

The distinct metabolic phenotypes of CD4 and CD8 T cells may promote specific functional responses. In particular, increased emphasis on glycolysis in CD8 cells may provide biosynthetic intermediates to promote rapid cell growth, while higher mitochondrial oxidative capacity of CD4 cells may allow the use of diverse fuels to efficiently generate ATP under metabolic stress. Prior to proliferation, T cell activation leads to a one-day lag phase in which lymphocytes grow in biomass [3]. Glycolysis has been proposed to promote cell growth and the increased proliferation of CD8 T cells may have been due to more rapid cell growth. To compare CD4 and CD8 T cell growth after initial activation, lymphocytes were stimulated and cell size was measured over time. Measurement of cell size by particle size analyzer (**Fig. 8**) showed an equivalent and rapid increase in cell volume within the first 4 h of stimulation. CD4 and CD8 T cells then remained similarly sized up to 12 h after activation, at which point both T cell populations resumed rapid growth. CD8 T cells, however, were significantly larger than CD4 T cells by 24 h.

**Figure 9:**
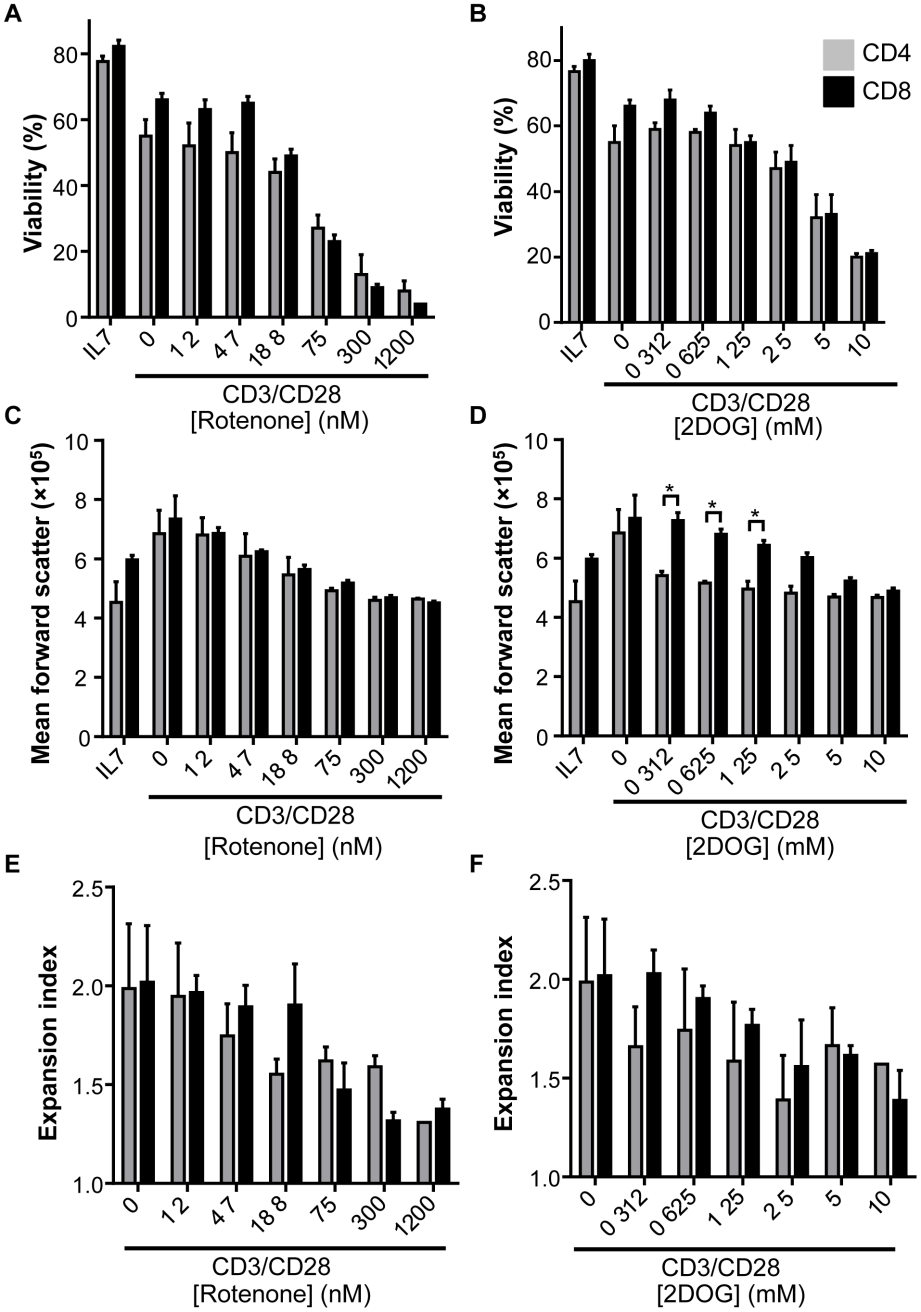
CD8 T cell growth is more resistant than CD4 T cell growth to glycolytic inhibition. CD4 and CD8 T cells were labeled with CellTrace Violet and activated for 48-CD3 anti-CD28 in the presence of indicated doses of rotenone or 2-deoxyglucose (2-DG). (**A, B**) Mean forward light scatter (cell size) of activated cells and (**C, D**) expansion index, (the fold expansion of the original culture) as determined by CellTrace violet dilution are shown. Data shown are representative of two experiments; data show mean ± standard deviation.

The increased oxidative capacity of CD4 T cells may allow greater metabolic flexibility. The ability of CD4 and CD8 T cells to grow and proliferate was, therefore, tested with inhibition of glycolysis or electron transport. Isolated CD4 and CD8 T cells were cultured in IL-7 alone to maintain viability or stimulated with anti-CD3 and anti-CD28 for 48 h in the presence of increasing concentrations of either the glycolysis inhibitor 2-deoxyglucose (2-DG) or the mitochondrial complex I inhibitor rotenone. The viability of CD4 and CD8 cells was similarly affected by increasing inhibitor dose (**Fig. 9A, B**). Flow cytometric measurement of forward angle light scatter to indicate cell size showed that CD4 growth was significantly more sensitive to the presence of 2-DG (**Fig. 9C**), with sub millimolar 2-DG reducing CD4 T cell growth. Rotenone had similar effects on both CD4 and CD8 cell growth (**Fig. 9D**). Proliferation was assessed by calculating the fold-expansion of surviving CTV-labeled cells (expansion index) [31]. 2-DG and rotenone each inhibited T cell proliferation in a dose-dependent manner, with expansion index decreasing as inhibitor dose increased. No significant difference in proliferative sensitivity to the inhibitors was detected between CD4 and CD8 cells, although there was a trend towards greater sensitivity of CD4 T cells (**Fig. 9E, F**). Together, these data demonstrate that in comparison to CD8 cells, CD4 growth is more sensitive to metabolic inhibition.

## Discussion

Naïve CD4 and CD8 T cells perform low rates of oxidative metabolism to maintain survival, perform immunosurveillance, and undergo homeostatic proliferation. After activation, both CD4 and CD8 T cells reprogram towards a highly glycolytic metabolic program, generating energy and biosynthetic materials for rapid growth, proliferation and differentiation. Here we show that CD4 and CD8 T cells share many metabolic characteristics but have key differences that may allow CD8 T cells to rapidly proliferate. Importantly, activated CD8 T cells had a more glycolytic metabolism than CD4 T cells, while CD4 T cells had higher rates of mitochondrial oxidative metabolism and a greater maximal respiratory capacity. Functionally, CD8 T cells grew and proliferated faster than CD4 T cells, and activation-induced growth of CD8 T cells was more resistant to glycolytic inhibition.

The finding that T cell subsets are metabolically distinct is consistent with a growing literature of metabolic state coordinating cell function and fate. Within the CD4 lineage, it was recently demonstrated that low doses of the mitochondrial ATP synthase inhibitor oligomycin could block activation-induced proliferation [32], suggesting a key role for mitochondrial ATP generation. CD4 T cells can also differentiate into functional subsets with effector or suppressor roles in immunity and inflammation. Importantly, we have previously shown that effector T cells, such as Th1, Th2, and Th17, are highly glycolytic, while regulatory T cells instead utilize mitochondrial oxidative pathways [2]. These T cell subsets require these pathways, and increased glycolysis of effector T cells may support rapid proliferation and inflammatory function, whereas oxidative metabolism of Treg may provide metabolic flexibility to function in diverse nutrient conditions. CD8 T cells have also been demonstrated to be highly glycolytic [6], [33]–[35], although the comparison with CD4 T cells has not yet been reported. These differential metabolic programs and requirements may allow CD8 T cells to enter and proliferate in tissues with limited nutrient availability.

While there remained differences between CD4 and CD8 T cells irrespective of activation context, mitochondrial mass and ROS production in each population was dependent on both the strength of the activating stimuli and cytokine context. Metabolic reprogramming is therefore partially dictated by the environment and by co-stimulatory signals. This is likely to be important *in vivo*, where T cells are presented with antigens of varied avidity in the presence of varied cytokine milieus, oxygen tensions, and nutrient environments. Recent studies using nutrient transporter knockout T cells [8], [12], [36] indicate that activation-induced metabolic reprogramming is critical for many inflammatory responses *in vivo*; however, metabolic comparison of *in vivo* reprogrammed CD4 and CD8 cells has not yet been performed. Metabolic inhibitors have been shown to suppress T cell responses in EAE, asthma, and graft versus host disease [2], [4], [37], [38], and a key goal of future work will be to determine how the *in vivo* activation environment alters metabolic reprogramming in specific disease states.

The signaling pathways that lead to distinct metabolic patterns in CD8 and CD4 T cells are unclear. T cell receptor expression and activation is similar in both subsets, suggesting that receptor proximal events are unlikely to lead to divergent metabolic programs. The metabolic reprogramming differences between these two populations may arise in part from subtle changes in the expression of key metabolic enzymes. CD4 T cells expressed more PKM2 than similarly stimulated CD8 cells. Pyruvate kinase is a central gatekeeper in directing the balance between oxidative and glycolytic metabolism [39], with reduced PK activity leading to poorer pyruvate generation and increased accumulation of glycolytic intermediates. PK activity is regulated by several mechanisms beyond isoform expression, including oligomerization, post-translational modification and localization [40]. CD4 T cells also had greater mitochondrial content than CD8 T cells and this increased with activation. Together these may support both enhanced mitochondrial oxidative metabolism in activated CD4 T cells and enhanced aerobic glycolysis in CD8 cells. CD8 T cells also appeared to have greater mTOR pathway activity. The mTOR pathway provides a strong stimulus for anabolic metabolism and glycolysis that characterized CD8 T cells [5].

Many cell types adopt aerobic glycolysis as a metabolic strategy to fuel proliferation [17]. The elevated glycolytic rate of activated CD8 T cells may, therefore, explain the slightly faster rates of proliferation and growth observed in these cells in both the current study and previous *in vitro* and *in vivo* comparisons [41]–[44]. Indeed, reversal of aerobic glycolysis *in vitro* or *in vivo* using the pyruvate dehydrogenase kinase inhibitor dichloroacetate [45], or the glycolytic inhibitor 2-DG [46], [47] can be sufficient to prevent T cell proliferation, suggesting both CD4 and CD8 lymphocytes adopt aerobic glycolysis to maximize proliferative ability. While both CD4 and CD8 T cells were similarly sensitive to rotenone, activation-induced CD4 growth was significantly more sensitive to 2-DG. This suggests that CD8 cells were better able to compensate for the loss of glucose metabolism, and is reminiscent of Glut1 null T cells, where the absence of Glut has a significant effect on CD4 Th, but has minimal impact on CD8 cytotoxic T cells [12]. In the absence of glucose, activated CD8 T cells were more able to oxidize glutamine, suggesting that while CD4 cells primarily use glucose as an oxidative fuel, CD8s are metabolically more flexible. CD8 T cell growth may, therefore, be more resistant to 2-DG as the cells are better able to switch to glutamine oxidation.

Collectively, the data presented here demonstrate that CD4 and CD8 T cells utilize distinct metabolic strategies to support specific functional demands. Following activation CD8 T cells had a higher glycolytic flux than CD4 cells, correlating with rapid cell growth. CD4 T cells also induced glycolysis upon activation, but had greater mitochondrial content and oxidative metabolism than CD8 T cells. These metabolic differences are likely fundamental to cellular functions and may provide new directions to selectively target or promote specific T cell subsets.

## Materials and Methods

### Ethics Statement

All experiments were approved by the Institutional Animal Care and Use Committee at Duke University Medical Center (Protocol A260-11-10) and strictly followed the National Institutes of Health recommendations cited in the Guide for the Care and Use of Laboratory Animals. The Duke University Medical Center animal management program is accredited by the American Association for the Accreditation of Laboratory Animal Care.

### Mice

Mice expressing endogenous levels of myc-epitope tagged Glut1 (*Glut1^myc^*) have been described previously [2]. C57B6/J mice were obtained from the Jackson Laboratories.

Mice were bred and maintained within barrier conditions in one room of the animal facility at Duke University Medical Center. Mice were housed in individually ventilated cages and supplied with reverse osmosis purified water by an automatic system. Bedding and caging were autoclaved, and food was irradiated or autoclaved. All cage changes were conducted under a HEPA filtered cage-changing station. Experiments were performed using tissues from male and female mice aged between 6 and 14 weeks of age, with tissues pooled from same-sex littermates where necessary. All mice were euthanized by gradual exposure to CO_2_ in a dedicated chamber, with euthanasia being confirmed by bilateral thoracotomy.

### T cell Isolation, Culture and Inhibitors

CD4+CD25- and CD8+ T cells were isolated from spleen and lymph nodes to ≥90% purity by magnetic bead negative selection (Miltenyi Biotec). Cells were cultured in RPMI 1640 (MediaTech) supplemented with 10% FBS (Gemini BioProducts), penicillin-streptomycin (Gibco), L-glutamine (Gibco), and 50 µM β-ME (Sigma). Where indicated, cells were maintained in a quiescent, viable state using 10 ng/ml IL-7 (eBioscience). Alternatively, cells were stimulated on plates coated with 10 µg/ml anti-CD3 (clone 2C11) and 10 µg/ml anti-CD28 (both eBioscience) in the presence of 20 ng/ml IL-2 (Novartis), with a starting density of 0.5–1.5×10^6^ cells/ml. Cell concentration and size were measured using a Z2 particle counter (Coulter Corp.). Where indicated cells were activated in the presence of 2-deoxyglucose (Sigma) or rotenone (Seahorse Bioscience).

### Flow Cytometry

To confirm isolation purity cells were labeled with anti-mouse CD4-eFluor 450, CD8- phycoerythrin. Cy5.5, and Thy1.2- fluorescein isothiocyanate (FITC) (all eBioscience). Exofacially tagged Glut1^myc^ was stained with mouse anti-myc (Millipore, clone 4A6) followed by rat anti-mouse IgG-PE (eBioscience). Mitochondrial content was determined by labeling cells with mitotracker green or Deep Red FM (200 nM; Invitrogen). Reactive oxygen species were assessed by labeling cells with dichlorodihydrofluorescein (10 µM DCF; Invitrogen). Mitochondrial membrane potential was assessed using tetramethylrhodamine ethyl ester (200 nM TMRE; Invitrogen). Cell cycle status was assessed by treating methanol fixed cells with RNAse and then labeling DNA with propidium iodide (50 µg/ml PI; Invitrogen). Viable cell number was determined flow cytometrically by propidium iodide exclusion (1 µg/ml PI). Proliferation was assayed by flow cytometry of carboxyfluorescein succinimidyl ester (5 µM CFSE; Molecular Probes) labeled cells. Alternatively, proliferation and viability were assayed simultaneously by co-staining cells with CellTrace Violet (1 µM; Invitrogen) and 1 µg/ml PI. Cell size was determined by assessment of the visible light forward scatter of PI-excluding cells. Data were acquired on a MacsQuant cytometer (Miltenyi Biotec) and analyzed using FlowJo software (TreeStar).

### Immunoblotting

Immunoblotting was performed as described previously [10]. Briefly, cell pellets were snap frozen and then lysed on ice for 1 h in buffer containing 1% Triton X100, 0.1% SDS, protease inhibitor cocktail (BDBioscience) and phosphatase inhibitors I and III (Sigma). Lysates were pre-cleared by centrifugation and protein content determined by bicinchoninic acid protein assay (Biorad). Proteins were separated on 10–20% SDS-PAGE gradient gels (Biorad). Blots were probed for hexokinase I (Millipore, MAB1532), hexokinase II (Abcam, ab3279), hexokinase III (Abcam, ab126217), pyruvate kinase M2 (Cell Signaling Technology, 3198), cytochrome c (BDBiosciences, 556433), small ribosomal subunit S6 (Cell Signaling Technology, 2217), phospho-Ser235/6 small ribosomal subunit S6 (Cell Signaling Technology, 2211), Glut1 (Abcam, ab115730), mitochondrial complexes II-V (Abcam, ab110413), followed by mouse- or rabbit-conjugated horseradish peroxidase (HRP) (Cell Signaling Technology). HRP-conjugated antibodies were detected by enhanced chemiluminescence detection (Thermofisher).

### Measurement of ECAR and OCR

Oxygen consumption rate (OCR) and extracellular media acidification rate (ECAR) were measured using a XF24 extracellular flux analyzer (Seahorse Bioscience), as described [48]. Cells were attached to XF24 plates using Cell-Tak (BDBioscience). OCR and ECAR were measured in unbuffered RPMI (Sigma) supplemented with 10 mM D-glucose (Sigma), 10 mM L-glutamine, and/or 10 mM sodium pyruvate, as indicated in the figure legends. Where indicated cells were treated with 0.09% dimethyl sulfoxide (DMSO) (Sigma), 1 µM oligomycin, 0.5 µM carbonyl cyanide p-trifluoromethoxyphenylhydrazone (FCCP), 1.5 µM antimycin A, 0.75 µM rotenone (all seahorse bioscience). OCR and ECAR values were normalized to live cell number.

### Statistical Analysis

Statistical analyses were performed with Prism software (GraphPad) by student’s T test or two-way ANOVA. Following ANOVA significant differences were identified by Šidák multiple comparisons test. Expansion index was calculated using FlowJo software (Treestar). Data shown are mean ± standard deviation, statistically significant results are indicated (* p<0.05).
